# Long-Term Physiological Adaptations Induced by Short-Interval High-Intensity Exercises: An RCT Comparing Active and Passive Recovery

**DOI:** 10.3390/jfmk9040229

**Published:** 2024-11-12

**Authors:** Mario Mauro, Bernardino Javier Sánchez-Alcaraz Martínez, Pasqualino Maietta Latessa, Sofia Marini, Stefania Toselli

**Affiliations:** 1 Department of Life Quality, University of Bologna, 47921 Rimini, Italy; mario.mauro4@unibo.it (M.M.); pasqualino.maietta@unibo.it (P.M.L.); 2 Department of Physical Activity and Sport, Faculty of Sport Science, University of Murcia, 30100 Murcia, Spain; bjavier.sanchez@um.es; 3 Department of Medicine and Aging Sciences, “G. d’Annunzio” University of Chieti-Pescara, 66100 Chieti, Italy; sofia.marini@unich.it

**Keywords:** body composition, exercise, gender, physical performance

## Abstract

**Background:** High-intensity interval training (HIIT) is one of the most debated methods involving several parameters that could be modulated, but the long-term adaptations it induces are still unclear. This investigation aimed to evaluate the efficacy of running and whole-body exercises with high-intensity (>80% heart rate) short intervals (30 s) in body composition and physical performance and compare the effects between groups with active (AR) or passive recovery (PR), both in males and females. **Methods**: Eighteen trained young adults (55.56% ♀) were randomly allocated to the PR (n = 9, 23.09 ± 2.56 years, 163.69 ± 9.88 cm, 68.96 ± 14.62 kg) or AR (n = 9, 22.05 ± 1.54 years, 170.61 ± 11.5 cm, 68.78 ± 12.45 kg) group. Both groups performed eight weeks of HIIT, with an equal progression, training, and volume load (TL: F = 1.55, *p* = 0.214; VL: F = 0.81, *p* = 0.505). Body fat (BF), fat-free mass (FFM), upper and lower limb fat (UFI, LFI) and muscle areas (UMA, LMA), handgrip strength (HGS), power (countermovement jump, CMJ), agility (5-0-5), and maximal oxygen consumption ($\dot{V}$O_2p_) were tested before and after treatments. **Results**: The proposed HIIT reduced BF by 9.57% and increased FFM by 2.09%. Females reported better adaptations in LMA (8.34 times higher than males), while both sexes’ upper limb mass distribution was better affected by PR (♀: UFI g = 1.851, 95% CI: 0.51, 3.14; ♂: UFI g = 2.456, 95% CI: 0.336, 4.487). Concerning conditioning, the protocol increased $\dot{V}$O_2p_ by 6.47%. Females showed better adaptations in CMJ (RR = 1.8), while males showed better adaptations in agility (RR = 3.76). The interaction effects were significant for PR females (right = +6.28%; left = +9.28%) and for AR males (right = +19.21%; left = +19.04%) in HGS. **Conclusions**: Short-interval HIIT with different exercise recovery types may be a practical solution in training where several physiological improvements are needed. Coaches and trainers can take advantage of the versatile nature of HIIT, relying on desired movement patterns and long-term responses in both male and female individuals.

## 1. Introduction

Factors leading to high morbidity and mortality during the COVID-19 pandemic have enhanced the worldwide interest in health and wellness [1]. To date, exercise for improved health has guided the projection of the fitness industry, and weight loss and body composition appear as top trends across the globe. Since 2014, High-Intensity Interval Training (HIIT) has been one of the most debated training modalities owing to its versatile and dynamic nature [2]. HIIT involves repeated short-to-long bouts of exercise punctuated by rest periods at intensities modulated through physiological responses such as heart rate (HR), blood lactate, velocity associated with peak oxygen consumption ($\dot{V}$O_2p_), or rating of perceived effort (RPE) [3,4,5]. The role of exercise intensity is derived from the concept that a large volume of moderate-intensity or a small volume of high-intensity training can elicit similar skeletal muscle adaptation [6]. When training is matched for volume, some metabolic enzyme mediators are greatly stimulated as the intensity increases [7,8], eliciting gene expression [9] and mitochondrial biogenesis [7,10], and inducing physiological adaptations such as increasing $\dot{V}$O_2p_ [11,12]. When training is matched at a high intensity, the volume increment also augments the mitochondrial content [7]. In addition, a single high-intensity sprint bout increased the plasma catecholamine and growth hormone (GH) concentration post-exercise in both males and females, with hormones involved in fat metabolism and muscle gain [13].

However, at least nine HIIT parameters such as the work and rest modality, intensity, duration, number and duration of the series, time between each series, and between-series recovery intensity [5] can be managed to induce different physiological stimuli. Typically, work intervals shorter than 15 s, known as sprint interval training (SIT), allow athletes to reach a higher percentage of maximal effort, eliciting anabolic power and neuromuscular stress [14,15], while longer intervals of up to two minutes could favour reaching $\dot{V}$O_2p_, improving BLa and oxidative tolerance [16], and increasing the time to exhaustion to sub-maximal effort [17]. A time series between 15 s and one minute aims to induce metabolic (O_2_ system) and neuromuscular responses [5]. Furthermore, the recovery intensity and duration could be key in HIIT adaptations. Passive recovery (PR) between long work intervals facilitated training at a higher power output, maintaining similar session RPE [18,19], whereas active recovery (AR) at a moderate intensity (40–60% $\dot{V}$O_2p_) was more effective in removing BLa during the session [20,21]. Differently, in short work intervals, AR elicited a greater total power peak and work cost than PR with a similar RPE [22]. Despite the physiological mechanisms related to an acute response in recovery intervals having been well investigated, there is a lack of evidence on the long-term adaptation induced by high-intensity exercises that combine short intervals and AR or PR [23]. Furthermore, age and sex anthropometrical and biological features may be considered confounders or effectors and account for analysing specific physiological responses [24]. For example, females are supposed to accumulate a lower concentration of blood lactate after a 30-s sprint session, which could be associated with reduced basal activities of lactate dehydrogenase and muscle phosphofructokinase than males [25,26]. It remains unclear how the HIIT long-term adaptation could affect sexes differently, and whether HIIT protocols may be administered interchangeably between females and males.

Although the origin of HIIT has been credited with running and skiing exercises [4], this training modality has also been adapted to other sports [27] and fitness [28]. Many studies have shown that HIIT using whole-body exercises is effective in improving cardiorespiratory fitness; body composition, such as fat mass (FM) and fat-free mass (FFM); and musculoskeletal fitness, such as strength and endurance, in healthy adults [29]. Similar acute [30] and long-term [31] responses appeared between whole-body and running-based high-intensity training. Still, fewer studies have investigated the effect of combined running and whole-body HIIT [32].

In light of these pieces of evidence, the main purpose of this study was to evaluate the efficacy and feasibility of a novel 8-week HIIT program that combines short-term intervals with running and whole-body exercises and understand whether moderate-intensity AR (~50% HR) and PR induce different changes in body composition and physical performance between trained younger male and female adults.

## 2. Materials and Methods

### 2.1. Experimental Design

**Figure 1:**
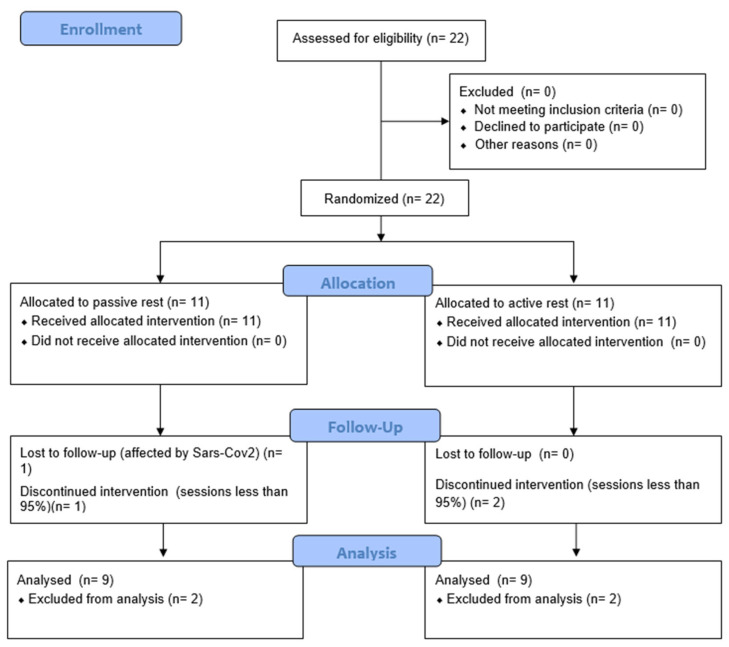
Consort flowchart.

A randomised clinical trial design of ten weeks was selected. The first (pre) and last (post) weeks were used to evaluate participants, whereas the HIIT treatments lasted eight weeks. Before the enrolment, a priori sample size was estimated for a repeated-measures analysis of variance test for within-between, following the study parameters: Type I error (α) = 5%, Type II error (β) = 20% and statistical power (1 − β) = 80%, number of groups and repeated measures = 4, variance between-within expected = 0.05 (Δ = 0.89), and correlation between repeated measures = 0.75. The estimated sample size was 20, four males and females for each group. To prevent the sample mortality effect, the sample size was increased by 10% (two subjects, Figure 1). This study was conducted in accordance with the Declaration of Helsinki, and approved by the Institutional Ethics Committee of the University of Bologna (protocol code 0058589, 3 March 2023). The trial is registered on ACTRN with the ID: ACTRN12624001352594 following the guidelines settled by CONSORT and ICMJE; https://www.anzctr.org.au/ACTRN12624001352594.aspx (accessed on 8 November 2024).

**Figure 2:**
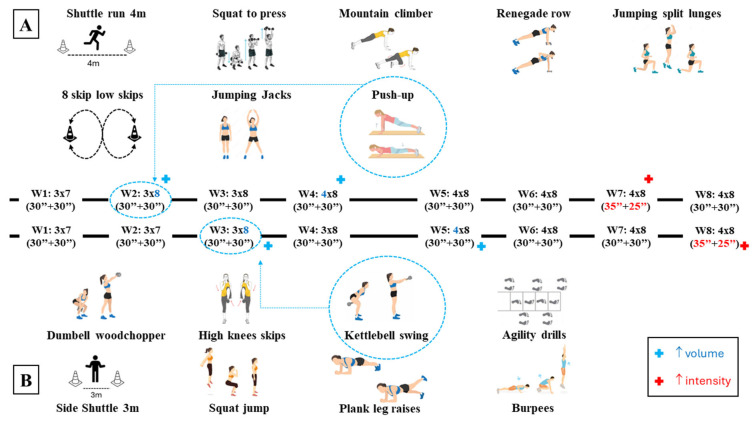
HIIT protocol exercises, parameters, and progression for session one (**A**) and session two (**B**) over eight weeks (W1–W8).

After registration, each participant was randomly allocated to AR or PR groups, which included the same exercises, series, work–rest ratio, duration, and progression (Figure 2). The randomisation was processed by a statistical software-specific package (STATA 18, Windows Edition, StataCorp, College Station, TX, USA). The AR protocol provided a walking recovery at 50% of maximal HR [22], whereas the PR group was requested to rest passively. Both HIIT programs included two weekly sessions, and participation in at least 15 (95%) workouts was needed. During each training session, the number of repetitions and loads per series, maximal and average HR (Polar H9 sensor and Polar beat mobile APP, Kempele, Finland), and rating of perceived effort (sRPE, 30 min after the workout end) were collected. Participants were tested for body composition, strength, power, agility, and maximal oxygen consumption before (pre) and after the HIIT treatment (post). The final performance evaluations were performed 72 h after the last HIIT session.

### 2.2. Participants

Eligibility criteria were as follows: (a) ages between 20 and 30 years old, (b) medical certification guarantee for high-intensity activities (exercise electrocardiography), (c) at least 5 years of adolescent sports experience with at least 2 training sessions per week during those years, and (d) no health problems, body limitations, or musculoskeletal injuries that could affect physical performance. Twenty-two subjects volunteered to participate in this study, but only 18 completed at least 95% of the training (Figure 1; soccer = 5, basketball = 1, volleyball = 1, swimming = 2, gymnastics = 5, boxing = 1, and athletics = 3). They were male (n = 8) and female (n = 10) university students (Faculty of Sports Science) who were fit (2.9 ± 0.84 sessions per week) and confident with the involved exercise techniques (Figure 1). Subjects were randomly allocated to PR (n = 9, 66.67% female, age = 23.09 ± 2.56 years, stature = 163.69 ± 9.88 cm, body mass = 68.96 ± 14.62 kg) or AR (n = 9, 44.44% female, age = 22.05 ± 1.54 years, stature = 170.61 ± 11.5 cm, body mass = 68.78 ± 12.45 kg). All subjects were asked to abstain from any other relevant physical activity or sport not included in the program provided. Also, subjects were asked to maintain their usual nutritional behaviours and avoid new dietary supplementation or drugs that could enhance body performance. Before the evaluations, each subject was instructed to have a two-week wash-out with no physical exercise.

### 2.3. Treatment

High-Intensity Interval Training. Figure 2A shows the first day and Figure 2B the second day of the training program for both AR and PR groups. Total time, session density, and work–recovery ratio followed previous recommendations [27]. The protocol included a short-term series (one minute) to elicit oxidative and neuromuscular system responses [5]. The series duration was maintained for the whole protocol, but the work–recovery ratio was modified to increase the training intensity over time. To induce mechanical tension, muscle damage, and peripheral metabolic stress, factors leading to muscle hypertrophy [33], each subject was asked to perform a maximal number of repetitions as possible (AMRAP) per series of each exercise, while they were asked to maintain 80–90% HR max during the work to increase central and peripheral oxidative demands. All the sessions were equal for groups regarding volume (number of exercises, series, and duration) and intensity (HR interval, work–recovery ratio, and rest between series). The groups differed by the recovery within the series during each training session. AR subjects were instructed to perform active recovery between series by walking at pre-tested speed (1.89 ± 0.26 m/s) in a specific gym rectangle (10 × 2 m) with marked lines for any metre. According to the session training rest time, they covered a specific distance related to their 50% HR max. Differently, the PR group had passive rest. Recovery time between each exercise (one minute) was passive for both groups. Standardised warm-up and cool-down were provided by one of the study investigators, who supervised each training session. In addition, he recorded in a logbook the number of repetitions (or laps) per series and exercise, the external load (if used), the averaged and maximal HR, and the RPE of the session. The training progression consisted of weekly increments in volume (weeks 2–5) and intensity (weeks 7–8) as follows: week 1 (W1) included three series of seven exercises for each work-out with a work ratio recovery of 1 (30 s:30 s, Figure 2) and total duration of 56 min (28 per session), week 2 added one exercise in work-out 1 (push-up, Figure 2A) for a week HIIT time of 60 min (32 and 28), W3 added one exercise in work-out 2 (kettlebell swing, Figure 2B) for a week time of 64 min, W4 increased one series in work-out 1 (4 series × 8 exercises) for a week time of 72 min (40 and 32), W5 increased one series in work-out 2 for a week time of 80 min, W6 had no increment to facilitate the adaptation, W7 changed the work–recovery ratio up to 1.4 (35 s:25 s) in work-out 1, and the last week increased the work–recovery ratio to 1.4 (35 s:25 s) in work-out 2.

#### Training and Volume Load

Before the HIIT program began, each subject was instructed to rate their perceived effort using a 0–10 scale [34], where 0–1 = very easy, 2 = easy, 3 = moderate, 4 = somewhat hard, 5–6 = hard, 7–8 = very hard, and 9–10 = maximal. Thirty minutes after the conclusion of each workout session, the investigator asked each participant to privately answer the question “How intense was your training?” and fill out the RPE 0–10 scale. The training load (TL) was computed for all sessions as the product of sRPE and the workout duration (minutes) [35]. Then, the adjusted training load (adj. TL) was calculated as follows:

$${(RPE}_{s}\cdot {time}_{s})\cdot \frac{{\bar{HR}}_{s}}{{HR}_{max}}$$

where s = session and ${\bar{HR}}_{s}=$mean of session HR.

In addition, the volume load (VL) was computed by multiplying the number of repetitions, the number of series, and the external load weighted (if used). Finally, the covered distance (CD) and the average speed (AS) were computed for each session by multiplying the number of repetitions for the metres provided for the exercise (CD), and then dividing it by the second of work. The TL was expressed in minutes, the VL was in kilograms, the CD was in metres, and the AS was in metres/seconds.

### 2.4. Anthropometry and Body Composition

Anthropometry and body composition evaluations were assessed on day one before (pre) and after (post) the HIIT protocol. Body mass (CCC = 1.000, 95% CI: 0.999, 1.000) was measured to the nearest 0.1 kg (Seca 769, Seca Scale Corp, Munich, Germany). Technical Error of measurement (TEM) = 3.18%. Arm (CCC = 1.000, 95% CI: 0.999, 1.000) and thigh (CCC = 0.999, 95% CI: 0.997, 1.000) circumferences were measured to the nearest 0.1 cm with no-stretchable tape (Seca, Seca Scale Corp., Munich, Germany), in standardised body sites [36]: the arm circumference was taken at the mid-point between the shoulder acromion and the olecranon process point, with the subject’s elbow relaxed along the body side, whereas the thigh circumference was taken at the mid-point between the inguinal fold and the superior kneecap point, with the participant in a standing position (thigh muscles relaxed). Arm and thigh TEM were, respectively, 2.01 and 1.25%. Arm and thigh muscle and fat mass areas were computed according to Lohman and colleagues [36]. Upper limb muscle area TEM = 5.29%, thigh muscle area TEM = 2.94%, while Upper limb fat index TEM = 4.56% and thigh fat index TEM = 5.61%. Triceps, abdomen, and thigh skinfold thicknesses were measured to the nearest 1.0 mm at the left side of the body (Lange, Beta Technology Inc., Houston, TX, USA), and then used to estimate body fat percentage according to Evans et al. [37]. The triceps site was marked vertically at the posterior arm face midpoint between the acromion process and the olecranon process; the abs site was marked horizontally three centimetres left and one above the umbilicus; the thigh site was marked vertically at the mid-point between the inguinal fold and the superior kneecap point. A trained investigator assessed the evaluations, and the average value of three repeated measures was used; their intraclass correlation coefficients (ICC) and random error of measurements (SEM) were: ICC = 0.948 (95% CI: 0.899, 0.978), SEM = 0.873 mm and TEM = 7.26%, ICC = 0.981 (95% CI: 0963, 0.992), SEM = 0.969 mm and TEM = 5.63%, and ICC = 0.988 (95% CI: 0.977, 0.995), SEM = 0.455 mm and TEM = 5.61%, for triceps, abs, and thigh, respectively. The predicted body fat, fat mass and fat-free mass reported a TEM of 5.34, 4.77, and 3.89%.

### 2.5. Handgrip Strength, Power, Agility, and Peak Oxygen Consumption

Handgrip strength (HGS), power (CMJ), and agility (5-0-5) were assessed on day two, while the peak oxygen consumption ($\dot{V}$O_2p_) was assessed on day three of the protocol in the first week and last week at the University Sports Science laboratory (Bologna, Italy). The indoor environmental features were 20 °C, 50–60% humidity, and no external music or soundtrack that could affect the performance, and they were unvaried among pre-and post-tests. Before the strength, power, and agility testing session, subjects performed a standardised warm-up, according to Bartolomei et al. [38]. For the maximal oxygen consumption, a standardised warm-up of five minutes of walking was assessed at the following speeds for each minute (1% inclination): 1.25 m/s, 1.39 m/s, 1.53 m/s, 1.67 m/s, and 1.81 m/s.

The handgrips strength for right (HGS r) and left (HGS l) hands were tested to the nearest 1 kg with an analogic dynamometer (Takei 5001, Takei Scientific Instruments Co., Ltd., Tokyo, Japan). Each subject stood with their arms by their sides and their elbows fully extended during evaluation. Three times of alternate measurements were made without a minute of rest among each series, and subjects were asked to squeeze the dynamometer for 3 s for each measurement [39]. The better result was used in the analysis. The HGS ICC were 0.983 (95% CI: 0.966, 0.993) and 0.980 (95% CI: 0.962, 0.002), while SEM was 1.315 and 1.434 kg and TEM was 4.56 and 5.14% for the right and left hand, respectively.

The countermovement jump (CMJ) test was assessed by a study investigator with photoelectric cells grounded at a two-metre distance (Optojump, Microgate, Bolzano, Italy). Subjects were instructed to maximise the height of each jump while keeping their hands on their hips. Flight time was calculated as the time interval from toe-off to landing. Each subject performed three jumps with a 2-min rest between each jump, and the best jump was used in the analysis. The CMJ intraclass correlation coefficient was 0.989 (95% CI: 0.978, 0.995), SEM = 0.909 cm, and TEM = 3.27%.

The 505 agility test was set up and administered using the protocol outlined by Draper [40]. Two investigators assessed the evaluation with two photoelectric cells connected to a digital chronometer (Witty SEM, Microgate, Bolzano, Italy) placed 10 and 15 m from the start line. Each subject was instructed to sprint after the acoustic signal for 15 m, turn on their preferred foot, and sprint back for another five metres. The time to cover the last five m of the 15 m straight line plus the 5 m after the change of direction was recorded. Three assessments with two minutes of rest between each series were performed. The best time was used for the analysis. The 505 agility test ICC was 0.892 (95% CI: 0.790, 0.954), SEM = 0.074 s, and TEM = 2.35%.

The treadmill Bruce test was set up according to Bruce protocol [41]. All subjects were asked to refrain from alcohol for 24 h prior and caffeine for 4 h before each trial. Also, subjects were asked to drink 500 mL of water approximately 2 h before testing to standardise body fluids concentration. Before the trial, each subject was attached to the safety vest and was instructed to push the stop button in case of emergency. Each subject performed a continuous incremental exercise test to voluntary exhaustion on a calibrated treadmill (h/p/cosmos pulsar, COSMED, Rome, Italy). A cardiac band for heart rate monitoring was provided (Polar H9 sensor, Polar, Kempele, Finland), and the entire trial was recorded by a mobile APP (Polar Beat, Polar, Kempele, Finland). The Bruce protocol consisted of incremental seven stages: (1) 3 min of walking with 10% inclination at 0.76 m/s, (2) 3 m of walking with 12% inclination at 1.12 m/s, (3) 3 m of walking with 14% inclination at 1.52 m/s, (4) 3 m of walking with 16% inclination at 1.88 m/s, (5) 3 m of running with 18% inclination at 2.24 m/s, (6) 3 m of running with 20% inclination at 2.46 m/s, and (7) 3 m of running with 22% inclination at 2.68 m/s. The trial ended when the subject was exhausted. The total length, average, and peak HR were collected. The Bruce equation was used to estimate the $\dot{V}$O_2p_. The TEM of predicted $\dot{V}$O_2p_ was 1.81%.

### 2.6. Statistical Analysis

For descriptive statistics, the mean was used as the central tendency measure, while the standard deviation was used for describing dispersion. The reliability of repeated measurements was computed as an intra-class correlation (ICC) and standard error of measurements (SEM) among baseline and follow-up, and as a relative technical error of measurements (TEM) over eight weeks.

To account for both within- and between-subjects correlation, the data were analysed, such as the preferred panel and the multivariate linear mixed effect model, where both fixed (mean model) and random (covariance model) effects were considered [42]. A full-way interaction of time, treatment, and gender was investigated. The same mean structure (fixed) was maintained by comparing three different covariance structures (unstructured, first-order autoregressive, and compound symmetry). The nested models (linear and quadratic) with different covariance structures were fitted by restricted maximum likelihood and compared throughout the likelihood ratio test. In addition, the Akaike information criteria (AIC) were checked for the best model. The normality assumption was checked for marginal residuals (Jacknifed studentised). When asymmetries in curves were found, a natural logarithm transformation was applied. To infer, the Wald test was assessed and the respective χ^2^ statistic with (n/2 − t) degrees of freedom was reported, where n is the sample size and t is the number of repeated measures. Also, the marginal effects were evaluated. The type I error probability was settled at 5%.

In addition, the percentage change was calculated as [(mean at post − mean at pre)/mean at pre] * 100. Where appropriate, the relative weighted change proportion was calculated as [(dependent var at post/weight var at post) − (dependent var at pre/weight var at pre)]/(dependent var at pre/weight var at pre). Finally, the effect size of the treatment was computed using the Hedges’ g statistic.

Data were gathered in digital spreadsheets in the Excel 2023 Windows edition (Microsoft, Washington, DC, USA) and analysed in STATA 18 Windows edition (StataCorp., TX, USA).

## 3. Results

### 3.1. Training Progression

Generally, the participants’ HR did not show significant changes over the eight weeks (79.34 ± 3.17 bpm, with a mean decrement of 0.54 bpm per week; z = −1.10, *p* = 0.272). When examined separately in the AR and PR groups and gender, the week progression did not significantly affect the HR variability (group: z = −0.82, *p* = 0.414; gender: z = −0.33, *p* = 0.74). However, the differences in the conditional means of AR vs. PR are statistically significant over each week, with a mean contrast of 4.45 ± 1.53 bpm (χ^2^_(8)_ = 20.24, *p* = 0.009), while female vs. male HRs differed only on W6 (χ^2^_(1)_ = 4.91, *p* = 0.027). The full-way interaction model showed significant differences in male AR vs. PR at W1 (β = 4.94 ± 2.31 bpm, χ^2^_(1)_ = 4.56, *p* = 0.033), in female AR vs. PR at W3 (β = 5.69 ± 2.04 bpm, χ^2^_(1)_ = 7.74, *p* = 0.005) and W8 (β = 5.35 ± 2.05 bpm χ^2^_(1)_ = 6.83, *p* = 0.009), and both sexes at W5 (♀: β = 4.33 ± 2.05 bpm, χ^2^_(1)_ = 4.49, *p* = 0.034; ♂: β = 6.96 ± 2.31 bpm, χ^2^_(1)_ = 9.05, *p* = 0.027), W6 (♀: β = 4.87 ± 2.05 bpm, χ^2^_(1)_ = 5.66, *p* = 0.017; ♂: β = 4.96 ± 2.31 bpm, χ^2^_(1)_ = 4.60, *p* = 0.032), and W7 (♀: β = 5.86 ± 2.05 bpm, χ^2^_(1)_ = 8.19, *p* = 0.004; ♂: β = 5.17 ± 2.31 bpm, χ^2^_(1)_ = 4.99, *p* = 0.026).

**Figure 3:**
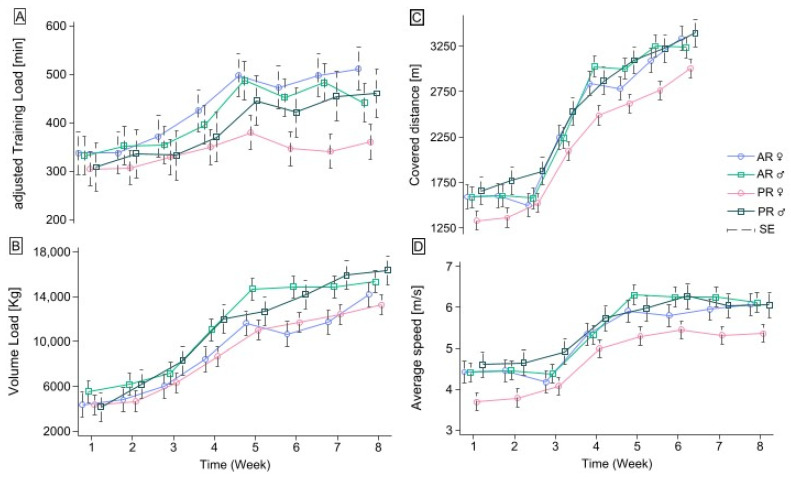
Training parameters’ variations over eight weeks. (**A**) Adjusted TL over weeks; (**B**) VL over weeks; (**C**) covered distance by participants over weeks and (**D**) its average speeds.

Figure 3 shows the adjusted TL (A), volume load (B), (C) coverage distance, and average speed (D). As regards the adjusted TL, the overall mean was 388.54 ± 100.84 min with an average week increment of 14.13 min (χ^2^_(7)_ = 145.77, *p* < 0.001). No difference appeared between AR vs. PR (z = 0.26 *p* = 0.794) and male vs. female (z = 1.61, *p* = 0.108), and adj. TL rates were significant for both groups (χ^2^_(14)_ = 165.87, *p* < 0.001) and sexes (χ^2^_(14)_ = 158.76, *p* < 0.001). When looking at marginals, the contrasts were significantly wider only in AR vs. PR females at W5 (β = 117.62 ± 56.77 min, χ^2^_(1)_ = 4.29, *p* = 0.038), W6 (β = 126.27 ± 56.77 min, χ^2^_(1)_ = 4.95, *p* = 0.026), W7 (β = 156.54 ± 56.77 min, χ^2^_(1)_ = 7.60, *p* = 0.005), and W8 (β = 151.12 ± 56.77 min bpm, χ^2^_(1)_ = 7.09, *p* = 0.008).

Figure 3B shows the VL means and trends for the groups and sexes. Generally, the VL reported a mean value of 9989.412, with a within-standard deviation of 3911.01 kg (r = 0.56). The baseline VL average was 3249.72 kg and the weekly effect affected it by 48.99% per week (z = 3.66, *p <* 0.001). The VL increments were not statistically significant just between weeks five and seven (W6 vs. W5 95% CI: −628.55, 1273.55; W7 vs. W7 95% CI: −186.82, 1715.27). Males reached a higher mean VL (β = 386.08 ± 194.69) than females (z = 1.98, *p* = 0.047), while no significant difference appeared between AR and PR over time (z = 0.64, *p* = 0.534). The interaction effect of the groups and sexes over time detected a constant trend (χ^2^_(16)_ = 12.64, *p* < 0.699).

Concerning the coverage distance (Figure 3C), the overall mean was 2346.82 ± 685.86 m. The weekly increment was 179.72 m (z = 4.98, *p* < 0.001), with a 9.30% rate higher in males compared with females (z = 2.11, *p* = 0.035); no differences appeared between AR and PR over time (z = 0.81, *p* = 0.419). The contrast of conditional predictions showed a significant difference between AR vs. PR females at W5 (β = 349.33 ± 167.36 m, χ^2^_(1)_ = 4.36, *p* = 0.037), W7 (β = 328.0 ± 167.36 m, χ^2^_(1)_ = 3.84, *p* = 0.05), and W8 (β = 334.67 ± 167.36 m, χ^2^_(1)_ = 4.0, *p* = 0.046).

Finally, the AS reported an overall mean value of 5.18 ± 0.77 m/s, with a baseline of 4.28 m/s and a weekly rate of 0.14 m/s (95% CI: 0.00, 0.29; *p* = 0.05). However, the marginal effects detected considerable changes at weeks four (95% CI: 0.80, 1.13) and five (95% CI: 0.37, 0.70). No significant differences appeared between the groups (z = −0.16, *p* = 0.875) and sexes (z = 1.84, *p* = 0.065) over time. The conditional contrast detected significant effects between AR and PR females at W1 (β = 0.73 ± 0.34 m/s, χ^2^_(1)_ = 4.53, *p* = 0.033) and W8 (β = 0.71 ± 0.34 m/s, χ^2^_(1)_ = 4.28, *p* = 0.039), while male speeds varied similarly.

### 3.2. Body Composition

**Figure 4:**
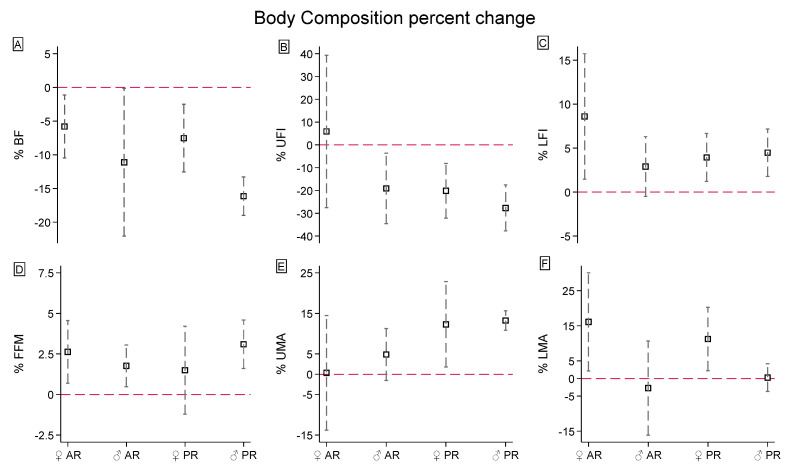
Body composition percentage changes after the 8-week HIIT protocol of (**A**) body fat, (**B**) upper-limb fat index, (**C**) lower-limb fat index, (**D**) fat-free mass, (**E**) upper-limb muscle and (**F**) lower-limb muscle areas. The red line is settled a 0% (no changes cutoff).

Table 1 shows the longitudinal effects of HIIT on body composition, stratified for groups and gender. Figure 4 shows the body composition changes (percentage) in AR and PR females and males. Table S1 reports the longitudinal effect sizes in body composition for each group and sex combination.

Eight weeks of HIIT reduced the %BF by 10.31 ± 2.4% (Figure 4A) from the baseline (95% CI: −15.50, −5.12), with no rate differences between the groups (+0.23% for PR, 95%CI: −1.06, 1.52) and genders (+1.15% for females, 95% CI: −2.56, 0.26). The conditional effects reported significant slopes for PR females (pre vs. post β = 1.60 ± 0.42%, χ^2^_(1)_ = 14.56, *p <* 0.001), PR males (pre vs. post β = 2.30 ± 0.59%, χ^2^_(1)_ = 15.01, *p <* 0.001), AR females (pre vs. post β = 1.29 ± 0.51%, χ^2^_(1)_ = 6.33, *p* = 0.012), and AR males (pre vs. post β = 1.28 ± 0.46%, χ^2^_(1)_ = 7.77, *p* = 0.005), with no differences in the rate of change (95% CI: −1.44, 2.46). However, the PR males reported the widest effect size (g = 2.583, 95% CI: 0.398, 4.683).

When observed peripherally, the UFI showed a 15.31 ± 21.27% decrement (Figure 4B). UFI marginal effects exhibited a greater variation in males (95% CI: −8.94, −0.47). Despite groups and their interaction with gender was not significant in mixed model rates (95% CI: −7.33, 4.33), the AR females showed a percentage UFI increment by 3.13% (95% CI: −5.78, 6.40; g = 0.136, 95% CI: −1.076, 1.336) while the PR females decreased by 20.13% (95% CI: −34.17, −7.31; χ^2^_(1)_ = 6.42, *p* = 0.01; g = 1.851, 95% CI: 0.507, 3.138). Differently, the mean LFI decreased by 4.77 ± 4.41% over time (Figure 4B). The slopes for groups and gender interactions were PR females pre vs. post β = 2.11 ± 0.74 (χ^2^_(1)_ = 8.21, *p* = 0.004; g = 0.31, 95% CI: −0.75, 1.355), PR males pre vs. post β = 2.53 ± 1.04 (χ^2^_(1)_ = 5.94, *p* = 0.015; g = 0.934, 95% CI: −0.532, 2.311), AR females pre vs. post β = 1.08 ± 0.90 (χ^2^_(1)_ = 1.43, *p* = 0.231; g = 0.157, 95% CI: −1.056, 1.358), and AR males pre vs. post β = 3.09 ± 0.81 (χ^2^_(1)_ = 14.74, *p* < 0.001; g = 0.754, 95% CI: −0.443, 1.91).

Concerning lean mass, FFM exhibited a mean increment of 2.09 ± 1.97% (Figure 4D). The marginal contrasts of AR females (β = −1.16 ± 0.54, 95% CI: −2.22, −0.11; χ^2^_(1)_ = 4.70, *p* = 0.03) and males (β = −1.24 ± 0.48, 95% CI: −2.18, −0.30; χ^2^_(1)_ = 6.68, *p* < 0.01), and PR males (β = −2.17 ± 0.62, 95% CI: −3.38, −0.95; χ^2^_(1)_ = 12.20, *p* < 0.001) reported significant slopes, whereas PR females were significantly unvaried (β = −0.70 ± 0.44, 95% CI: −0.70, 0.44; χ^2^_(1)_ = 2.54, *p* = 0.11). When observed peripherally, the UMA and LMA increased by 7.74 ± 10.31% and 6.26 ± 12.79% (Figure 4E,F), respectively. The marginal effects reported gender differences over time, which are detectable on UMA: males β = 4.90 ± 1.27 (95% CI: 2.40, 7.40; χ^2^_(1)_ = 14.76, *p* < 0.001) vs. females β = 1.94 ± 1.13 (95% CI: −0.26, 4.15; χ^2^_(1)_ = 2.98, *p* = 0.085). However, the gender and group interaction reported a significant slope in the PR females (β = 3.95 ± 1.43; χ^2^_(1)_ = 7.67, *p* = 0.006; g = 0.377, 95% CI: −0.689, 1.425). This discrepancy diverged for LMA, where PR (β = 4.28 ± 1.81, 95% CI: 0.73, 7.82; g = 0.536, 95% CI: −0.547, 1.593) and AR (β = 5.77 ± 2.22, 95% CI: 1.43, 10.11; g = 0.578, 95% CI: −0.69, 1.801) females’ and AR males’ (β = 7.54 ± 1.98, 95% CI: 3.65, 11.42; g = 0.606, 95% CI: −0.568, 1.745) increments were statistically significant, whereas PR males’ were not (χ^2^_(1)_ = 1.18, *p* = 0.277; g = 0.162, 95% CI: −1.129, 1.434).

### 3.3. Physical Performance

**Figure 5:**
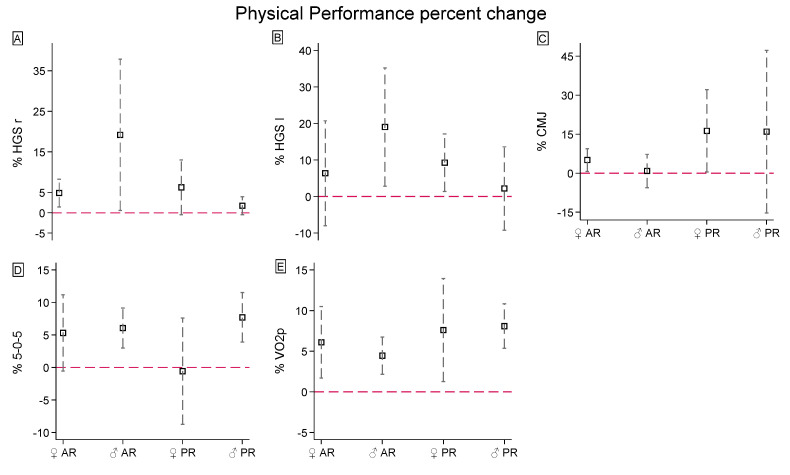
Physical performance percentage changes after the 8-week HIIT protocol of (**A**,**B**) right and left handgrip strength, (**C**) countermovement jump, (**D**) agility test and (**E**) Peak oxygen consumption. The red line is settled a 0% (no changes cutoff).

Table 2 shows the longitudinal effects of HIIT on the physical performance parameters, stratified for groups and gender. Figure 5 shows the physical performance changes (percentage) in AR and PR females and males. Table S2 reports the longitudinal effect sizes in the body composition for each group and sex combination.

Generally, only the maximal oxygen consumption appeared to be significantly affected by the HIIT protocol (6.47 ± 4.42% from baseline, Figure 5E), with different slopes for group and gender interactions: PR females (β = 3.06 ± 0.74, 95% CI: 1.61, 4.51; χ^2^_(1)_ = 17.11, *p* < 0.001; g = 0.737, 95% CI: −0.372, 1.812), PR males (β = 3.68 ± 1.05, 95% CI: 1.63, 5.74; χ^2^_(1)_ = 12.40, *p <* 0.001; g = 0.618, 95% CI: −0.656, 1.847), AR females (β = 2.62 ± 0.91, 95% CI: 0.84, 4.39; χ^2^_(1)_ = 8.34, *p* = 0.004; g = 0.701, 95% CI: −0.696, 2.024), and AR males (β = 2.25 ± 0.81, 95% CI: 0.66, 3.84; χ^2^_(1)_ = 7.71, *p* = 0.006; g = 0.46, 95% CI: −0.694, 1.587).

Regarding handgrip strength and power, when looking at marginal effects, the AR protocol improved by 19.21 ± 18.64% right (β = 7.4 ± 1.81, 95% CI: 3.84, 10.96; χ^2^_(1)_ = 16.63, *p <* 0.001; g = 0.715, 95% CI: −0.476, 1.866) and by 19.04 ± 16.18% left HGS (β = 6.5 ± 1.97, 95% CI: 2.63, 10.36; χ^2^_(1)_ = 10.85, *p* = 0.001; g = 0.487, 95% CI: −0.671, 1.616) in males (Figure 5A,B), whereas the PR protocol increased by 16.26 ± 15.8% CMJ in females (β = 3.87 ± 1.67, 95% CI: 0.59, 7.14; χ^2^_(1)_ = 5.36, *p* = 0.021; g = 1.024, 95% CI: −0.132, 2.136). The CMJ also positively increased in AR females (g = 0.375, 95% CI: −0.861, 1.582) by 5.06 ± 4.36% (Figure 5C).

In addition, the HIIT protocol enhanced agility (Figure 5D) in male participants of PR by 7.70 ± 3.81% (β = 0.30 ± 0.14, 95% CI: 0.03, 0.57; χ^2^_(1)_ = 4.80, *p* = 0.029; g = 1.605, 95% CI: −0.114, 3.22) and AR by 6.05 ± 3.07% (β = 0.25 ± 0.11, 95% CI: 0.04, 0.43; χ^2^_(1)_ = 5.37, *p* = 0.020; g = 0.991, 95% CI: −0.251, 2.182), with an increasing rate of 72.88% compared to females (χ^2^_(1)_ = 8.44, *p* = 0.004).

## 4. Discussion

The main aim of the following investigation was to evaluate the efficacy of an 8-week HIIT program with both running and whole-body exercises in improving body composition and physical performance in trained young adults. We found that two HIIT sessions of about 36 min per week with short intervals (~30 s) and a work–recovery ratio of ~1 positively affected body health. The protocol was HR-based with %HR ranging from 80 to 100% in work intervals [5]. During the follow-up, both the volume (number of exercises, series, and total duration) and intensity (work–recovery ratio) were gradually increased to induce the best long-term adaptations [6,7,11]. Although the group with active recovery reached higher average HR values of 8.48 ± 2.88 bpm, the sex and group characteristics did not affect the HR’s variability rate over the eight weeks. This suggests that the proposed protocol maintained a comparable HR trend for both groups and active recovery helps to elicit the cardiovascular system widely. Also, the mentioned physiological pattern was more evident during the last four weeks, pointing to greater human sensitivity in the training intensity. It is in line with Plews and colleagues [43], who demonstrated that variations in the training load may influence the HR responses. To evaluate both the training load and HR variation, we adjusted it by the HR mean and max ratio and detected a weekly increment of 3.65%, with a linear trend in AR and PR males and females. This accounted for steeper slopes between weeks four and five, where the protocol saw the bigger change in volume, and six and seven where participants performed greater changes in intensity. This also reflects the volume load variation, which rapidly increased between weeks three and five, where 71.32% of its increment was covered. According to Granata and colleagues [7], when training is settled at a high intensity, a higher volume could increment the activity of citrate synthase, the protein content of electron transport system subunits, PGC-1α, NRF1, TFAM, PHF20, and p53. In addition, when the maximal amount of the total volume suggested for HIIT is reached, a further increment in intensity could elicit peripheral adaptations such as a higher rate of glycogen utilisation, and the greater activity of AMPK, CaMKII, and ATF2 [9]. All these markers of the mitochondrial content and transcription factors are involved in mitochondrial biogenesis and can modify cellular energy requirements. In fact, due to the mitochondrial density regulating the substrate metabolism during submaximal exercise, a greater muscle enzyme and protein content could promote fat oxidation than glycogen degradation [6]. The above-mentioned physiological mechanisms accord with our results in body compositions induced by the HIIT protocol progression. Although longer intervals (>2 min) could favour triglyceride depletion due to a wider amount of time spent in the oxidative metabolism, our protocol positively affected the subjects’ total body fat by 10.31%, showing that the increasing total volume and intensity are effective even if shorter intervals have performed. This finding is in line with Macpherson and colleagues [44] who found that 18 SIT sessions decreased body fat by 6.4%, with a 1% increase in lean mass. However, if we focus on exercise selection, to the best of our knowledge, just two studies assessed a combination of whole-body exercise with short intervals, and the investigators did not find improvement in the relative fat mass and fat-free mass [32,45]. Although the subjects trained three times per week in both investigations, Eather and colleagues [32] subministered sessions that lasted from 8 to 12 min, which could not be enough to elicit fat oxidation, while the HIIT protocol of Evangelista et al. [45] lasted six weeks and it lacked progression within the weeks. The doubled weekly session duration of our protocol (~30 min vs. ~70 min per week) suggested that two workouts per week are effective when the volume is appropriate.

Interestingly, the reduction in upper limb adiposity, such as its increment in the muscle area, accounted for the great body composition improvement, whereas the lower limb fat increased. Female participants exhibited a general increment in the lower limb area (+13.19% muscular and +5.81% fat), while the upper limb better varied in both sexes. Differently, males worsen their lower limb mass partitioning. The observed sex differences may be justified by evidence suggesting females possess a greater predisposition for aerobic metabolism, due to a bigger (~10%) relative Type I fibre area [46], with an oxidative contribution 25% higher than males during short-interval exercise (≤30 s) [47]. The sex difference occurs during HIIT recovery periods where females exhibited smaller ATP and faster restoration [25]. Also, a similar higher response in the muscular area has been detected by Esbjirnsson and colleagues [25] who found that females had a greater increase in the type-II fibre cross-sectional area than males after 30 s of SIT. So, the well-stated greater muscle oxygen delivery in females may lead to a higher muscle glycogen content and lipid metabolism [46]. The results we found agree with Hazell and colleagues [48] who demonstrated that six weeks of sprint interval training with short work intervals (30 s) improved the body mass distribution (−8% FM, +1.3% FFM) in active women. Despite Trapp and colleagues [49] stating that HIIT with short work intervals induced a total body fat decrement, they found a wider reduction in lower than upper limbs. This discrepancy with our results can be explained by differences in the training protocol since participants in the previously mentioned study performed just cycling exercises.

Concerning the recovery type, PR is akin to the AR group in terms of body composition. According to previous evidence, active recovery at ~50% HR could not be enough to impair the energy balance and induce a wider oxygen debt [18]. Compared to active rest at 80% or 110% of the lactate threshold during long interval bouts, a shorter work–rest ratio at 50% HR does not appear to affect blood lactate fasting and the related perceived effort. When interacting with sex, we found that passive recovery improved the female upper area better than AR. The two groups reported different slopes in the weekly volume load, covered distance, and average speed, with a constant positive trend in passive recovery and some flatness in active recovery females (weeks one to three and four), which could have enhanced the mechanical cost and heat release. However, no previous study has compared PR and AR between males and females to definitively state long-term adaptations, so further investigations are needed.

As previously mentioned, modulating parameters (volume, density, intensity, etc.) involved in high-intensity interval exercise have been effective in stimulating both peripheral and central adaptations such as an increased maximal blood and stroke volume, cardiac output, and other factors related to physical capacity [6]. Several studies found significant improvements in acute and long-term $\dot{V}$O_2p_ after the HIIT protocol [11]. Our results found that eight weeks with 16 sessions of HIIT were effective in increasing $\dot{V}$O_2p_ when progression is well monitored and the HR-based intensity ranges are close to the planned cut-off values. When approximatively matched for the interval duration, our findings accord with Astorino et al. [50] who evidenced how shorter bouts widely affected $\dot{V}$O_2p_. In addition, a recent meta-analysis provided systematic evidence on how the high volume (≥15 min per session) and moderate-to-long term (4–12 weeks of protocols) could ensure the greatest $\dot{V}$O_2p_ improvements in healthy adults [51]. However, females reported the biggest effect sizes similar for PR (+7.6%) and AR (+6.1%), whereas PR males increased by 81.90% compared to AR. Previous studies have shown that active recovery in long-interval training (>2 min) favours reaching and maintaining the VO_2max_ threshold, enhancing the metabolic responses [52]. A rationale physiological consequence of the daily metabolic peak reached could be followed by positive long-term adaptations in $\dot{V}$O_2p_. No previous studies, nevertheless, have found similar results comparing active and passive recovery in adaptive outcomes, and evidence on acute responses shows that time at $\dot{V}$O_2p_ ≥ 80% did not differ in recovery at several intensities [18]. Differently, it is well-stated that males accumulate more blood lactate after 30 s of repeated sprints, with a lower level of aerobic contribution, which could lead to downstream signals that regulate muscle adaptations [24]. As a direct consequence, due to ~25 s of recovery corresponding to the minimal time at which no lactic acid accumulation took place [4], males could prefer passive recovery to allow partial metabolic restoration that contributes a longer time to exhaustion, higher speed, and wider distance covered. Accordingly, the weekly CD and AS trends for PR males presented higher slopes that explain a constant increase in running parameters (supposed to greatly affect $\dot{V}$O_2p_).

Although HIIT benefits on $\dot{V}$O_2p_ have been well-stated in both athletic and healthy adults, the same could be figured out on strength, power, and agility just in competitive athletes. Stankovic and colleagues [53] found that HIIT is a time-effective approach to moderately improve the explosive strength tested by CMJ in female volleyball, soccer, and basketball adult players, whereas the agility measured by the change in the direction shuttle test was widely affected. We supposed combining running and whole-body exercise could enhance strength and power adaptations [29]. In addition, about 30 s of work trying to perform as many repetitions as possible could initially promote fast fibre-type recruitment increasing intra-muscular coordination, and then the major eliciting slow-fibre type increasing inter-muscular coordination [54], followed by improvements in the handgrip strength, power, and agility. However, previous evidence has suggested that “all out” bouts with long recovery is the best solution to reach the peak of power or speed because the fully restoring substrate reserves allow for performing the maximal neuro-muscular effort, while long intervals are favoured to reach the maximal oxygen consumption and lactate tolerance [3]. The last two statements rationally lead to planning exercise protocols with short work intervals and long recovery, but less of a contribution has been given to maximal oxygen consumption. In light of this, we implemented a protocol with whole-body and running exercises and short intervals to promote a full range of physical improvements. In terms of handgrip strength, power, and agility, we found that the proposed protocol with combined exercise affected the sexes and groups differently. The passive rest protocol greatly affected the power adaptive response in females (+16.26%) and males (+15.97%) and strength responses in females (+7.78%) than the AR counterpart. Also, despite AR males showing a good increment in speed (+6.05%), PR males reported a 1.27 times higher change. According to previous results, we found that the wider metabolic restoration elicits power, strength, and agility improvements. Interestingly, the active recovery enhanced changes in HGS males (9.67 times higher than PR males) and 5-0-5 females (9.15 times higher than PR females). To the best of our knowledge, this study is the first study that investigated how physiological adaptations differ between active and passive recovery males and females in longitudinal high-intensity training that combines running and whole-body exercises, and it makes it difficult to report a direct comparison. Several studies discussed the benefits of HIIT protocols on strength, power, and agility, but the effects of the recovery type on males and females need more attention.

This study reported some limitations: (a) the sample size could have negatively affected the type I error probability, reducing the statistical effects; (b) the four participants who dropped out impartially divided the groups for the gender (PR reports a higher ratio of females); (c) fatigue was measured only by RPE without accounting for the blood lactate concentration.

## 5. Conclusions

This study demonstrated that 8 weeks of well-monitored HIIT with two sessions per week and combined exercise decreases body fat and increases fat-free mass in young trained adults. Performing a combination of whole-body and running exercises included in a short-interval protocol with the work–recovery ratio near the unit is effective for conditioning both males and females, especially in terms of maximal oxygen consumption. The PR, nevertheless, is suggested for improving lower limb power and female strength, while AR is more appropriate for agility and male strength. The use of different recovery types may be a practical solution in sports where the main goal is training closer to the maximal oxygen consumption threshold, but other parameters need to be elicited. Strength and conditioning trainers should be aware of the dynamic nature of HIIT, which allows them to select appropriate exercises and modulate several variables for reproducing the metabolic requirements of a specific sport in both male and female competitions. The possibility of inducing physical adaptations with less than two hours per week of HIIT makes its utilisation optimal for each training periodisation phase.

## Figures and Tables

**Table 1:** Longitudinal HIIT effects on Body Composition in groups.

PR (n = 9)					AR (n = 9)				Mixed Model Effects (Wald Test, *χ*^2^ Degrees of Freedom)							
	Pre	Post	Pre	Post	Pre	Post	Pre	Post								
	♀ (66.67%)		♂ (33.33%)		♀ (44.44%)		♂ (55.56%)		Time		Time/Group		Time/Gender		Time/Group/Gender	
	Mean (±SD)	Mean (±SD)	Mean (±SD)	Mean (±SD)	Mean (±SD)	Mean (±SD)	Mean (±SD)	Mean (±SD)	z	*p*	z	*p*	z	*p*	z	*p*
BF (%)	21.65 (1.79)	20.05 (2.30)	14.16 (0.93)	11.86 (0.38)	20.83 (3.01)	19.54 (2.10)	11.04 (3.29)	9.76 (2.88)	−3.09	0.002 *	0.35	0.723	−1.59	0.111	0.51	0.607
UFI (%)	37.41 (4.03)	29.65 (3.70)	28.56 (0.87)	20.71 (3.50)	31.65 (5.28)	32.64 (7.24)	24.33 (7.03)	20.36 (8.92)	−1.51	0.132	0.56	0.580	−2.18	0.029 *	−0.50	0.614
LFI (%)	27.15 (6.36)	25.04 (6.21)	20.16 (2.18)	17.62 (2.16)	26.63 (5.55)	25.55 (6.37)	15.57 (3.45)	12.48 (3.93)	−2.23	0.026 *	0.78	0.437	−1.01	0.314	−1.14	0.253
FFM (kg)	48.44 (7.41)	49.14 (7.39)	71.18 (10.56)	73.35 (10.62)	45.23 (1.84)	46.39 (1.02)	69.41 (7.20)	70.65 (7.58)	1.40	0.161	0.62	0.537	2.22	0.026 *	−1.30	0.193
UMA (cm^2^)	35.67 (9.83)	39.62 (9.49)	53.29 (12.97)	60.15 (13.31)	33.02 (2.98)	32.96 (4.05)	51.61 (13.28)	54.56 (16.37)	2.30	0.022 *	−1.84	0.066	1.94	0.052 *	0.06	0.952
LMA (cm^2^)	70.54 (7.00)	74.82 (7.73)	89.95 (13.93)	92.73 (13.43)	65.62 (8.55)	71.38 (8.77)	88.01 (8.95)	95.55 (13.13)	1.84	0.065	0.14	0.887	0.74	0.457	0.81	0.416

Note: n, sample size; PR, Passive Recovery; AR, Active Recovery; F, Snedecor–Fisher test; *p*, *p*-value; SD, Standard Deviation; BF, Body Fat; FFM, Fat Free-Mass; UFI, Upper limb Fat Index; LFI, Lower limb Fat Index; UMA, Upper limb Muscle Area; LMA, Lower limb Muscle Area; *, statistically significant.

**Table 2:** Longitudinal HIIT effects on physical performance in groups.

PR (n = 9)					AR (n = 9)				Mixed Model Effects (Wald *χ*^2^ Degrees of Freedom)							
	Pre	Post	Pre	Post	Pre	Post	Pre	Post								
	♀ (66.67%)		♂ (33.33%)		♀ (44.44%)		♂ (55.56%)		Time		Time/Group		Time/Gender		Time/Group/Gender	
	Mean (±SD)	Mean (±SD)	Mean (±SD)	Mean (±SD)	Mean (±SD)	Mean (±SD)	Mean (±SD)	Mean (±SD)	z	*p*	z	*p*	z	*p*	z	*p*
HGS r (kg)	31.00 (4.00)	32.83 (3.82)	49.17 (11.03)	50.17 (12.17)	31.63 (2.69)	33.13 (2.25)	41.20 (8.87)	48.60 (9.79)	0.25	0.804	0.03	0.977	3.11	0.002 *	−0.73	0.464
HGS l (kg)	28.67 (4.93)	31.17 (4.54)	46.17 (14.89)	46.17 (11.62)	30.88 (3.42)	33.00 (6.48)	40.10 (13.25)	46.60 (10.71)	0.68	0.496	−0.09	0.926	2.20	0.027 *	−0.93	0.354
CMJ (cm)	23.97 (2.48)	27.83 (4.25)	36.97 (9.90)	41.40 (8.05)	28.58 (2.99)	30.05 (3.77)	40.32 (10.05)	40.16 (8.06)	0.38	0.706	0.03	0.973	1.21	0.226	0.56	0.573
Agility (m/s)	3.89 (0.42)	3.84 (0.14)	3.98 (0.18)	4.28 (0.11)	3.81 (0.32)	4.01 (0.26)	4.08 (0.19)	4.33 (0.26)	1.42	0.154	−1.15	0.251	1.72	0.085	0.64	0.525
VO_2peak_ (mL/kg#min)	42.70 (4.42)	45.77 (3.17)	46.38 (4.43)	50.07 (3.96)	45.26 (4.37)	47.87 (2.79)	51.48 (4.51)	53.73 (4.32)	2.89	0.004 *	−0.10	0.917	0.77	0.438	0.21	0.832

Note: PR, Passive Recovery; AR, Active Recovery; z, statistical test z; *p*, *p*-value; SD, Standard Deviation; HGS r, Handgrip Strength right; HGS l, Hand Grip Strength left; CMJ; Countermovement Jump; VO2, maximal oxygen consumption; *, statistically significant.

## Data Availability

The dataset is free—available on 10.5281/zenodo.13627555.

## Supplementary Materials

The following supporting information can be downloaded at: https://www.mdpi.com/article/10.3390/jfmk9040229/s1, Table S1: Edge’s longitudinal effect sizes in body composition; Table S2: Edge’s longitudinal effect sizes in physical performance.
