# Supplementary material for: Long-Term Physiological Adaptations Induced by Short-Interval High-Intensity Exercises: An RCT Comparing Active and Passive Recovery
Source: J Funct Morphol Kinesiol. 2024 Nov 12;9(4):229. doi: 10.3390/jfmk9040229 (PMC11587179; doi:10.3390/jfmk9040229)
Supplement: Supplementary file 1 [file jfmk-09-00229-s001.zip › jfmk-3241056-supplementary.pdf]

Table S1. Edge's longitudinal effect sizes in body composition.

	PR fem	AR fem	PR mal	AR mal
	g (95% CI)	*g* (95% CI)	*g* (95% CI)	*g* (95% CI)
BF (%)	0.716 [-0.389, 1.789]	0.432 [-0.812. 1.642]	2.583 [0.398, 4.683]	0.374 [-0.771, 1.496]
UFI (%)	1.851 [0.507, 3.138]	0.136 [-1.076, 1.336]	2.456 [0.336, 4.487]	0.446 [-0.706, 1.573]
LFI (%)	0.31 [-0.75, 1.355]	0.157 [-1.056, 1.358]	0.934 [-0.532, 2.311]	0.754 [-0.443, 1.91]
FFM (kg)	0.087 [-0.96, 1.13]	0.677 [-0.608, 1.913]	0.163 [-1.128, 1.435]	0.151 [-0.975, 1.268]
UMA (cm^2)	0.377 [-0.689, 1.425]	0.015 [-1.19, 1.218]	0.417 [-0.914, 1.7]	0.179 [-0.949, 1.295]
LMA (cm^2)	0.536 [-0.547, 1.593]	0.578 [-0.69, 1.801]	0.162 [-1.129, 1.434]	0.606 [-0.568, 1.745]

Table S2. Edge's longitudinal effect sizes in physical performance.

	PR fem	AR fem	PR mal	AR mal
	g (95% CI)	*g* (95% CI)	*g* (95% CI)	*g* (95% CI)
HGS r (kg)	0.432 [-0.639, 1.482]	0.525 [-0.733, 1.744]	0.069 [-1.213, 1.342]	0.715 [-0.476, 1.866]
HGS l (kg)	0.487 [-0.59, 1.54]	0.355 [-0.878, 1.561]	0.0 [-1.277, 1.277]	0.487 [-0.671, 1.616]
CMJ (cm)	1.024 [-0.132, 2.136]	0.375 [-0.861, 1.582]	0.392 [-0.934, 1.673]	0.016 [-1.134, 1.104]
Agility (m/s)	0.147 [-0.902, 1.19]	0.596 [-0.675, 1.822]	1.605 [-0.114, 3.22]	0.991 [-0.251, 2.182]
VO_{2peak} (ml/kg#min)	0.737 [-0.372, 1.812]	0.618 [-0.656, 1.847]	0.701 [-0.696, 2.024]	0.46 [-0.694, 1.587]
